# Progressive Additive Benefits of Prehabilitation and Subsequent Bariatric Surgery on Cardiac Autonomic Regulation as Assessed by Means of a Simple Unitary Composite Index: Preliminary Data from an Observational Study

**DOI:** 10.3390/jpm12081317

**Published:** 2022-08-15

**Authors:** Luca Giovanelli, Carlo Palombo, Matteo Pina, Simone Facchetti, Mara Malacarne, Massimo Pagani, Monica Nannipieri, Rossana Berta, Daniela Lucini

**Affiliations:** 1 BIOMETRA Department, University of Milan, 20129 Milan, Italy; 2 Exercise Medicine Unit, Istituto Auxologico Italiano, IRCCS, 20135 Milan, Italy; 3 Department of Endocrine and Metabolic Medicine, Istituto Auxologico Italiano, IRCCS, 20149 Milan, Italy; 4 Department of Surgical, Medical, Molecular Pathology and Critical Area Medicine, University of Pisa, 56126 Pisa, Italy; 5 Department of Clinical and Experimental Medicine, University of Pisa, 56216 Pisa, Italy; 6 Obesity Surgery Division, Pisa University Hospital, 56216 Pisa, Italy

**Keywords:** bariatric surgery, lifestyle intervention, autonomic nervous system, heart rate variability, exercise, sympathetic activity, prehabilitation

## Abstract

Obesity is associated with an increased risk of several chronic comorbidities, which may also be determined by dysfunctional autonomic nervous system (ANS). The influence of bariatric surgery (BS) on ANS balance was explored in previous studies, but with high heterogeneity in both the assessment timing and methods employed. In the present observational study, we applied a clinical protocol which considers two subsequent phases. Twenty-nine non-diabetic obese subjects were studied at baseline (T0), after one month of lifestyle modification (prehabilitation) (phase 1-T1), and after eight months following BS (phase 2-T2). ANS regulation was assessed across the three study epochs by means of ANSI, a single composite percent-ranked proxy of autonomic balance, being free of gender and age bias, economical and simple to apply in a clinical setting. The aim of the present study was to investigate the effects of the clinical protocol based on prehabilitation and subsequent BS on the ANS regulation by means of ANSI. Potential intertwined correlations with metabolic parameters were also investigated. Notably, we observed a progressive improvement in ANS control, even by employing ANSI. Moreover, the reduction in the markers of sympathetic overactivity was found to significantly correlate with the amelioration in some metabolic parameters (fasting glucose, insulin levels, and waist circumference), as well as in stress and tiredness perception. In conclusion, this study provides convincing evidence that a unitary proxy of cardiac autonomic regulation (CAR) may reflect the progressive improvement in autonomic regulation following behavioral and surgical interventions in obese patients. Intriguingly, this might contribute to reducing cardiovascular and metabolic risk.

## 1. Introduction

Obesity is associated with an increased risk of several chronic comorbidities, such as type 2 diabetes, arterial hypertension, myocardial infarction, and cancer [1,2,3]. The mechanisms underpinning this association are complex and multifarious. In addition to immunological and hormonal alterations, there is compelling evidence supporting a meaningful role of dysfunctional autonomic nervous system (ANS) in this context [4,5,6]. Obese subjects of any age are indeed characterized by ANS impairment, owing to a dominant activity of the sympathetic component over the vagal one [7,8,9,10,11], which ultimately leads to structural and functional abnormalities in the cardiovascular system [12]. This autonomic dysregulation is present both in resting conditions and in response to physiological stressors [13]. Actually, the relationship between obesity/insulin resistance and autonomic dysfunction seems to be bidirectional, featuring a circular causality [14,15]. The elevation of insulin and inflammatory markers levels—related to the abdominal fat excess—may in fact contribute to sympathetic activation, which in turn reduces insulin sensitivity, thus promoting both the onset and progression of organ damage [7,16].

Management of obesity is first predicated on lifestyle modification, which induces functional and structural improvements by modulating hemodynamic load as well as hormonal and immunological profiles, with a final favorable impact on the cardio-metabolic risk. Notably, gains in ANS regulation might also play a relevant complementary part in this setting [4]. In fact, long lasting interventions based on physical exercise and/or healthy nutrition have been shown to reverse—at least partially—autonomic impairment in patients with several chronic conditions, such as arterial hypertension, ischemic heart disease, and diabetes [7,17,18,19,20,21]. According to a recent feasibility study from our group [22], betterments in cardiac autonomic phenotype (i.e., combination of evidence of increased vagal flow and decreased sympathetic drive) may be achieved even after short periods of lifestyle interventions in candidates for bariatric surgery (BS). Interestingly, we also observed a strong connection between changes in insulin levels and in autonomic activity [22].

In order to optimize the outcomes of bariatric surgery, adhering to a lifestyle program based on physical activity and calorie-restricted diets is usually recommended [23,24,25,26] before surgery. Bariatric surgery has been correlated with a 90% improvement in obesity-related comorbidities as well as an up to 0.5% decrease in mortality [23,24]. Undeniably, the adoption of a healthy lifestyle should precede the surgical procedure, in order to improve the outcome, and should be embedded in everyday life following BS, in order to reduce weight and maintain weight loss in the long term [25,26,27].

Previous studies have explored the influence of BS on ANS balance [28]. All of the different types of BS performed (Roux-en-Y gastric bypass, vertical sleeve gastrectomy, biliopancreatic diversion, with or without duodenal switch, and adjustable gastric band) proved to be effective in improving heart rate variability (HRV). In some cases, this increase in HRV was positively correlated with a decrease in insulin resistance and leptin levels [29,30,31]. Diabetic patients experienced earlier betterments in the ANS control markers as compared to their non-diabetic counterpart [30,32]. Notably, exercise training was seen to sustain or even enhance the benefits of BS on the cardiac autonomic regulation (CAR) [12]. Furthermore, increased parasympathetic activity following BS could, in turn, contribute to mitigate food intake [33]. However, there was high heterogeneity among studies both in the HRV assessment timing (ranging from 7 days to 12 months after BS procedure) and methods employed (e.g., Holter electrocardiograms, plethysmography, Polar RS800CX HR monitor, VariaCardio T4 device, and echocardiography). Moreover, most of these techniques are tricky to carry out and interpret.

In this respect, it would be crucial to find simple non-invasive methods to evaluate and monitor CAR. For instance, the Autonomic Nervous System Index (ANSI) represents a single composite percent-ranked proxy of autonomic balance, whereby higher values indicate better autonomic control [34,35,36]. ANSI is, by design, free of age and gender bias, and correlates with cardiac baroreflex sensitivity, which is considered as an important cardiac prognostic predictor [37].

The aim of the present study was to investigate the effects of a clinical protocol based on prehabilitation and subsequent BS on the Cardiac Autonomic Regulation (CAR) by means of ANSI. Potential intertwined correlations with metabolic parameters were also explored. Indeed, the increased risk of cardiovascular events triggered by autonomic dysfunction in obesity turns the spotlight on the importance of monitoring autonomic function with simple but reliable methods, with a view to deploying therapeutic strategies that aim to restore autonomic balance [38,39].

## 2. Materials and Methods

Twenty-nine subjects, aged 45.2 ± 9.9 years and affected with morbid obesity (BMI ≥ 40 kg/m^2^) in the presence of a normo-tolerant glucose status, were enrolled at the Obesity Surgery Unit of Pisa University Hospital five weeks before undergoing bariatric surgery.

Eligibility criteria for obesity surgery followed international guidelines [40], recommending surgery on top of diet in III degree obesity without comorbidities and in II degree obesity combined with comorbidities. Patients with diabetes or established hypertension were excluded from the study. Additional exclusion criteria were: pregnancy, body-weight change >5 kg in the 3 months preceding the screening, abnormal thyroid hormone levels or any other possible cause of “secondary” obesity, immunodeficient conditions or anemia, use of vitamin and mineral supplementation within 3 months of screening, use of medication affecting body weight, energy expenditure or glucose control, or antibiotic treatment in the last month, drug or alcohol abuse, and drinking of >3 cups of coffee daily. Participants experiencing nausea, fever, vomiting, bloody diarrhea or severe abdominal pain, or having had cardiovascular, cerebrovascular, kidney, or liver diseases during the last 6 months before the beginning of the present study were also excluded. Participants were first evaluated when enrolled (T0). After a 4-week lifestyle intervention program (prehabilitation), based on low calories balanced diet and non-supervised physical activity, they were examined again (T1) [22]. Hence, they underwent bariatric surgery (Roux-en-Y gastric bypass 71.4%, vertical sleeve gastrectomy 28.6%), and they were reassessed 8.4 ± 4.0 months after procedure (T2).

### 2.1. Program Outline

- Clinical assessment (necessary also to eligibility for bariatric surgery) comprised the following
  - History, evaluation of previous medical tests, standard medical examination;
  - Routine blood tests: Serum total cholesterol, HDL-cholesterol, LDL-cholesterol, triglycerides (Roche Method for Modular Systems, Basel, Switzerland); glucose (Cobas Integra -Roche, Basel, Switzerland), insulin (determined by a specific time-resolved fluoro-immunoassay-Auto DELFIA Insulin kit; Wallac Oy, Turku, Finland), alanine transaminase(ALT), aspartate transaminase (AST) and gamma-glutamyl-transferase (GGT), and Creatinine, determined using standard clinical methodology employed at Pisa University Hospital;
  - Anthropometric (height, weight, Body Mass Index (BMI), Waist circumference (WC));
  - Resting metabolic rate (RMR) was estimated by indirect calorimetry [41] which was performed by a computerized open-circuit system with a canopy (Vmax 29 N; SensorMedics, Yorba Linda, CA, USA).
- Cardiac Autonomic Regulation (CAR)

On the day of autonomic evaluation, all subjects arrived at the clinic avoiding caffeinated beverages since awakening as well as heavy physical exercise in the preceding 24 h. Recordings were performed between 09:00 am and 12:00 am in an air conditioned, quiet room. After a preliminary 10-min rest period in the supine position, ECG and respiratory activity (piezoelectric belt) were continuously recorded over a minimum 5-min period with a two-way radiotelemetry system (Marazza, Monza Italy). Subsequently, subjects were asked to stand up unaided and remained in the upright position for 5 min, during which recordings were maintained. Data were acquired with a PC at 250 samples/second using a custom built software tool (HeartScope) that automatically provided a series of indices describing Heart Rate variability (HRV) in the time domain: RR interval (in msec) and RR interval Variability (assessed as total power, i.e., variance, in msec^2^, taken as simple classifiers typical of vagal control [36,42,43]; and in the frequency domain: autoregressive spectral components both in the low frequency (LF, center frequency ≈0.1 Hz) and in the high frequency (HF, centered with respiration, ≈0.25 Hz), assessed in msec^2^ as well as in normalized units (nu). To include an approximate evaluation of the effects of sympathetic activation, produced by active standing, the stand-rest difference (Δ) in LFnu was also computed.

Arterial pressure was continuously monitored using a plethysmographic approach (Finometer Pro, FMS) calibrated against a sphygmomanometer and analyzed simultaneously with the same software tool. The sensitivity of spontaneous cardiac baroreflex control of RR interval was assessed by a frequency-domain method (Alpha-Index = square root of the average of the ratio between RR interval and SAP Spectral powers in the LF and HF regions) [37] usable as a bivariate metaclassifier.

Recently, to simplify clinical interpretation of multiple HRV variables, we described a unitary autonomic index (ANSI) [36]. Computation of ANSI depends on the combination of principal factor analysis and clinically optimized radar plot, considering the cardiac autonomic information carried by RR, RR interval variance and Δ LFnu [36]. The computing procedure first corrects for age by percentile rank transformation; second, ranks the information (82.7% of Variance Accounted For) distributed across indices from the selected clusters of variability (considering amplitude and oscillatory code modalities [36,37]; and third, using a radar plot, builds ANSI as a composite [44] triangle area that is finally per cent ranked against the benchmark population. ANSI is treated as a proxy of cardiac autonomic regulation.

- Lifestyle assessment
  An ad hoc questionnaire to quantify lifestyle [45,46,47,48,49,50] was employed
  - Routine blood tests: Serum total cholesterol, HDL-cholesterol, LDL-cholesterol, triglycerides (Roche Method for Modular Systems, Basel, Switzerland); glucose (Cobas Integra-Roche, Basel, Switzerland), insulin (determined by a specific time-resolved fluoro-immunoassay-Auto DELFIA Insulin kit; Wallac Oy, Turku, Finland), alanine transaminase(ALT), aspartate transaminase (AST) and gamma-glutamyl-transferase (GGT), Creatinine, determined using standard clinical methodology employed at Pisa University Hospital. Physical activity (total activity volume) was assessed by a modified version of the commonly employed short version of International Physical Activity Questionnaire (IPAQ) [49,50], which focuses on intensity (nominally estimated in Metabolic Equivalents (MET) according to the type of activity) and duration (in minutes) of physical activity. We considered the following levels: brisk walking (≈3.3 METs), other activities of moderate intensity (≈4.0 METs) and activities of vigorous intensity (≈8.0 METs) [48];
  - Nutrition was assessed using the Healthy Diet Score [48], taking into consideration fruit/vegetables, fish, sweetened beverages, whole grain, and sodium consumption (the assessment of this latter one was adapted to Italian eating habits [47];
  - Stress and somatic symptoms perception were assessed using a self-administered questionnaire [45,46,47] providing nominal self-rated scales (higher values indicate higher degrees of symptoms) that focused on: (i) the appraisal of overall stress and fatigue perception by Likert linear scales from 0 (‘no perception’) to 10 (‘highest perception’) for each measure; (ii) The Subjective Stress-related Somatic Symptoms Questionnaire (4S-Q), inquiring about 18 somatic symptoms accounting for the majority of somatic complaints. For scoring purpose, each response was coded from 0 (‘no feeling’) to 10 (‘a strong feeling’), thus the total score ranged from 0 to 180. The protocol of this study followed the principles of the Declaration of Helsinki and Title 45, US Code of Federal Regulations, Part 46, Protection of Human Subjects, Revised 13 November 2001, effective 13 December 2001, and was approved by the local Institutional Ethics Committee (Study protocol N. 140/2014, approved on 13 March 2014). As per policy of Pisa University Hospital all the Subjects gave their written informed consent to participate in the research study and to allow publication of anonymized data.

### 2.2. Statistical Analysis

Data are presented as mean ± SD (Standard Deviation). Differences between data at T0, T1, and T2 were assessed with repeated measures General Linear Model, considering *p* < 0.05 as significance threshold. Individual contrasts were assessed with the Bonferroni correction. Spearman correlations and Linearity Test (LT) were also employed. Computations were performed with a commercial package (IBM SPSS 27).

## 3. Results

### 3.1. Anthropometric, Metabolic and Hemodynamic Parameters

Table 1 reports changes in anthropometric and metabolic parameters, as well as in blood pressure. As expected, a significant reduction in weight, BMI, WC, and systolic arterial pressure (SAP) was observed both at T1 (after 1 month of prehabilitation) and T2 (8.4 ± 4.0 months after bariatric surgery). Fasting glucose and insulin levels significantly declined after BS, with an improvement in oral glucose insulin sensitivity (OGIS) and HOMA index. A significant change in insulin levels and HOMA index was also detected at T1. Resting metabolic rate was significantly lower at T2 compared to both baseline and T1. Further blood tests, encompassing HbA1c, lipid profile, liver and kidney function, were performed only at T0 and T2 due to cost restrictions. Notably, significant variations in HbA1c (38.75 ± 4.99 and 34.28 ± 3.29 mmol/mol, respectively; *p* = 0.004), triglycerides (112.94 ± 45.43 and 89.25 ± 15.58 mg/dL, respectively; *p* = 0.03), and GGT values (24.34 ± 10.75 and 14.72 ± 6.49 U/L, respectively; *p* = 0.001) were observed. No significant differences were noted regarding other blood tests; in particular: creatinine (0.75 ± 0.17 and 0.75 ± 0.11 mg/dL, respectively; *p* = 0.4), total cholesterol (187.40 ± 34.87 and 182.75 ± 21.13 mg/dL, respectively; *p* = 0.2), HDL cholesterol (54.38 ± 11.97 and 53.44 ± 7.76 mg/dL, respectively; *p* = 0.9), LDL cholesterol (114.66 ± 31.79 and 106.47 ± 28.48 mg/dL, respectively; *p* = 0.2), AST (20.5 ± 6.55 and 20.47 ± 5.92 U/L, respectively; *p* = 0.5), and ALT (25.66 ± 12.38 and 23.05 ± 17.81 U/L, respectively; *p* = 0.4).

### 3.2. Lifestyle Questionnaire

**Figure 1:**
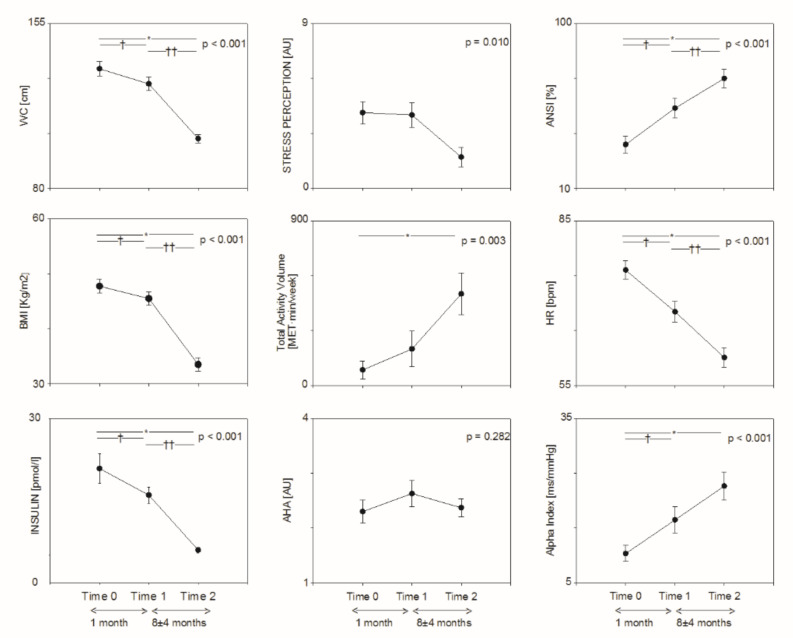
Changes in metabolic (**left** panels), lifestyle (**middle** panels) and autonomic (**right** panels) parameters from baseline (Time 0) to Prehabilitation (Time 1) and after bariatric surgery (Time 2). * T2 vs. T0: *p* < 0.05. † T1 vs. T0: *p* < 0.05; †† T2 vs. T1: *p* < 0.05.

Total physical activity volume (see Table 2 and Figure 1) was progressively increased after BS (*p* < 0.001 from LT), while AHA nutrition score was slightly improved after prehabilitation, but not after BS. No significant changes were observed in sedentariness. Perception of stress and tiredness were significantly reduced after BS.

### 3.3. Cardiac Autonomic Control Variables

As shown in Table 3 and Figure 1, heart rate was progressively reduced (*p* < 0.001 from LT), while total RR interval variability was progressively increased (*p* < 0.001 from LT). Both ANSI (single composite percent-ranked proxy of autonomic balance, whereby higher values indicate better autonomic control) and Alpha Index (a proxy of baroreflex sensitivity) were progressively increased (*p* < 0.001 from LT). The high frequency component of RR interval variability (HFRR [nu]) was greater at T2 as compared to T0.

### 3.4. Correlations

Additionally, by considering changes between T0 and T2, the reduction of the LF component of RR interval variability (marker of prevalent sympathetic activity to the SA node) was found to significantly correlate with the improvement in stress (r = 0.460; *p* = 0.014) and tiredness (r = 0.483; *p* = 0.009) perception, as well as in some metabolic parameters, encompassing fasting glucose (r = 0.843; *p* < 0.001), insulin levels (r = 0.467; *p* = 0.024), and WC (r = 0.554; *p* = 0.003). We also observed a significant correlation between weight decrease and improvement of several parameters, such as HR (r = 0.430; *p* = 0.010), RR LF/HF ratio (r = 0.537; *p* = 0.001), stress (r = 0.523; *p* = 0.004) and tiredness (r = 0.572; *p* = 0.001) perception, fasting glucose (r = 0.525; *p* = 0.037), HbA1c (r = 0.805; *p* < 0.001), insulin levels (r = 0.761; *p* < 0.001), HOMA index (r = 0.753; *p* < 0.001), and RMR (r = 0.705; *p* < 0.001). Moreover, the increase in Alpha-Index correlated significantly with the reduction in fasting glucose concentrations (r = 0.564; *p* = 0.023). As expected, there was a significant correlation between the decrease in SAP and in some metabolic parameters, including BMI (r = 0.377; *p* = 0.028), WC (r = 0.409; *p* = 0.043), fasting glucose (r = 0.578; *p* = 0.019), HbA1c (r = 0.612; *p* = 0.007), insulin (r = 0.416; *p* = 0.048), HOMA index (r = 0.422; *p* = 0.045), and RMR (r = 0.608; *p* = 0.003).

## 4. Discussion

In this investigation we observed a progressive improvement of Cardiac Autonomic Regulation in a group of non-diabetic obese patients studied at baseline (T0), after one month of prehabilitation based on lifestyle modification (T1), and eight months after Bariatric Surgery (T2). This improvement was clear even by employing ANSI, a combined proxy of ANS function which may overcome some pitfalls of the non-invasive ANS evaluation, comprising the high number of parameters derived from spectral analysis of cardiovascular variabilities, age and gender influences, and costs/difficulties linked to the need for continuous recording of systolic arterial pressure. In addition, by considering changes between T0 and T2, the reduction in the LF component of RR interval variability (marker of prevalent sympathetic activity to the SA node) was found to significantly correlate with the improvement in some metabolic parameters (fasting glucose, insulin levels and WC) and with the reduction in stress and tiredness perception.

### 4.1. Overweight, Obesity and Autonomic Nervous System

Nowadays, bariatric surgery represents a prominent strategy in the management of severe obesity [23], leading to a sizeable reduction not only of BMI, but also (and more importantly) of cardiovascular and metabolic risk. In fact, obesity and overweight increase the risk for arterial hypertension, coronary artery disease, heart failure, diabetes, and other diseases, such as cancer, and osteomuscolar and chronic lung disorders [1,2]. Recent data also show a role of obesity in worsening COVID-19 prognosis [51]. Overweight and obesity are characterized by an impairment of ANS prior to the onset of diabetes or cardiac disease [3,5,6,52,53], thus suggesting a possible role of ANS alongside other etiopathogenetic mechanisms in the development of these conditions. Of particular interest is the relationship between insulin resistance and ANS control in obese patients. Overweight, obesity, and insulin resistance are strongly associated with an increased risk of several chronic diseases, ranging from myocardial infarction to diabetes and cancer [1,2,3]. The processes underlying this association are complex and multifarious, and there is compelling evidence supporting the potential role of an altered autonomic function (i.e., dominant activity of the sympathetic component over the vagal one) in addition to humoral (or inflammatory) alterations [4,5,6]. From a pathophysiological perspective, whether ANS dysregulation is the consequence or the cause of obesity and insulin resistance remains matter of debate [14,15]. Autonomic changes—particularly sympathetic overactivity—may reduce insulin sensitivity and also favor the onset and progression of organ damage both directly and indirectly [16]. On the other hand, it is difficult to pinpoint the exact mechanisms behind these autonomic alterations. Overall, data indicate a possible coexistence of multiple neurohumoral mechanisms activating the sympathetic nervous system. For instance, ANS dysregulation may be triggered by impaired reflex mechanisms such as baroreflex, increased psychological stress, augmented oxidative stress and/or inflammation [3], and sympathetic overactivity. These latter processes are tightly coupled with reduction in adipokines [54] and increase in other inflammatory mediators [55] fostered by excessive abdominal fat, eventually resulting in insulin resistance. On the other hand, elevated insulin levels may induce sympathetic activation [7], in a circular causality.

Clinical intervention to manage obesity based on lifestyle improvement (physical activity and healthy nutrition) and subsequently bariatric surgery, as suggested by guidelines [23,40,56], represent a pivotal strategy to improve simultaneously several control mechanisms such as metabolic, immunological (inflammatory), and autonomic controls, which are intertwined. The reduction in adipose tissue (which is mild in the first phase of intervention based on lifestyle modification and, subsequently, dramatic in the second phase based on BS) may lead to an amelioration of all those control mechanisms. In particular the improvement in metabolic control after BS may lead to better cardiac autonomic regulation. In the present paper, we observed a progressive improvement in autonomic control close to a progressive reduction in BMI, WC, and insulin levels across the three study epochs. Moreover, the reduction in the markers of prevalent sympathetic activity from T0 to T2 well correlated with the reduction in fasting glucose, insulin levels and WC, hence suggesting a parallel improvement in multiple control mechanisms following weight reduction.

### 4.2. Autonomic Nervous System Assessment in Clinical Practice

The study of ANS in a clinical setting may represent an important diagnostic tool, in order to identify early alterations preceding full-blown diseases and/or to highlight improvement following weight reduction interventions. In line with our previous findings revealing a correlation between ANS dysfunction and metabolic alterations [52,53,57], in the present study, we observed a progressive improvement of ANS regulation following both smaller and greater weight reduction (after one month-prehabilitation and BS, respectively). To the best of our knowledge, this progressive improvement (LT *p* < 0.001 for both ANSI and Alpha Index) has been never observed hitherto. Indeed, previous papers showed post-BS increase in parasympathetic activity, not always combined with decrease in insulin resistance [28], by employing nonetheless invasive techniques [7,58,59] or 24-h Holter recordings [28,60,61]. The heterogeneity in the methodologies used to analyze HRV, as well as in the HRV parameters considered, may—at least partially—explain the conflicting data regarding the effects of BS on CAR. In contrast, in the present study, we assessed ANS control using a convenient, non-invasive technique [36,37,42], which provides time and frequency domain components of heart rate/systolic arterial pressure variability by means of few minutes recording only. In particular, the most informative HRV indexes [37] were employed to build ANSI, a unitary proxy of CAR, being free of gender and age bias, simple to apply in a clinical setting, and integrating information on both supine rest and standing up. Further, the use of a percent ranking permits an individual evaluation within the reference benchmark population. In summary, ANSI attributes a percent ranked (0–100) measure of CAR, whereby higher values indicate better autonomic control. Notably, ANSI, which is built by considering only HRV variables, was also found to correlate with cardiac baroreflex (Alpha Index) in a relatively large general population [37], with potential economic and organizational advantages derived from the lack of continuous blood pressure monitoring. All these properties render ANSI particularly useful, economical and suitable to gauge the results of individual clinical intervention such as prehabilitation and BS.

### 4.3. Bariatric Surgery, Stress and Lifestyle Modification

Lifestyle modification plays a pivotal part in the management of cardiometabolic conditions. In candidates for BS it can act as an adjuvant strategy to improve patients’ fitness and reduce surgical risk. Both in the present and in a previous paper on a larger population [22], we showed that one month of lifestyle-based prehabilitation program ameliorated metabolic parameters and CAR, and that a further intervention involving BS led to a further improvement of such outcomes. Lifestyle modification can have a crucial role after BS as well [62], being not only essential to maintain some of the benefits achieved, but also to determine some additional advantages [27]. In particular, physical exercise is a key factor to sustain metabolic gains [27] and to grant greater improvement in lipid and glucose profile after surgery [63]. A study conducted on obese adolescents proved a relationship between exercise and increased insulin sensitivity regardless of the impact of BS [64]. CAR improvement induced by endurance physical exercise is a further prominent benefit alongside those linked to weight reduction [4,17,18,65]. Moreover, physical exercise helps to counteract some undesirable effects of BS, including increased bone turnover and decreased muscle mass and function [27]. Exercise may contribute to prevent or minimize RMR decline during weight loss [66], especially in bariatric patients [67]. It is also worthwhile taking into account that bariatric patients do not always follow healthy lifestyle recommendations after surgery [63]. In a previous study on a larger population, AHA nutrition index increased significantly after prehabilitation [22]. On the other hand, in the present paper, we noticed that this index was unmodified eight months after surgery as compared to baseline, despite a slight improvement during prehabilitation. On the contrary, the endurance exercise volume progressively increased across the three study epochs, thus suggesting an important contribution of exercise to the obtained beneficial results. The small sample size did not allow to perform statistical analysis capable of detecting to what extent post-surgery weight loss and physical training, respectively, determined CAR improvement. Notably, stress is an independent risk factor for cardiometabolic diseases [68], particularly for severe obesity [69]. In this study, participants reported a reduced perception of stress and tiredness after BS, which suggests a potential further mechanism behind the reduction of cardiometabolic risk in these patients, acting both directly—by improving immunological, hormonal, and autonomic controls—and indirectly—by facilitating adherence to healthy lifestyle (especially physical activity).

### 4.4. Limitations

Several limitations must be considered. We studied only 29 patients. However, despite the small sample size, intertwined autonomic and metabolic benefits were found. Some blood tests, encompassing HbA1c, lipid profile, and liver and kidney function, were performed only at T0 and T2 due to cost restrictions. Indeed, we decided to focus on CAR, being the effects of prehabilitation and bariatric surgery on metabolic parameters already well known. We considered patients who underwent two different BS procedures: Roux-en-Y gastric bypass and vertical sleeve gastrectomy. We enrolled all the eligible subsequent patients of Obesity Surgery Unit of Pisa University who accepted to participate to the study. The specific BS procedure was decided by surgeon considering patients’ clinical characteristics. Other preliminary study conducted by other groups [28,29,30,31] showed that different types of BS performed were effective in improving heart rate variability, suggesting that the main mechanism responsible of this effect may, plausibly, be linked to the loss of fat mass more then to the different surgical procedure.

## 5. Conclusions

This observational study provides significant evidence that a unitary proxy of CAR may easily and conveniently reflect the progressive improvement in autonomic regulation following behavioral and surgical interventions in obese patients.

Although these findings are only preliminary, they can support the growing amount of data showing an instrumental role of autonomic nervous system improvement in the reduction of cardiovascular and metabolic risk following clinical interventions.

## Figures and Tables

**Table 1:** Descriptive statistics of entire population at the three study epochs.

Variables	Time 0	Time 1	Time 2	Significance
***n***	29	29	29	
**Smoke [%]**	24.1			
**Age [years]**	45.2 ± 9.9			
**Height [cm]**	164.7 ± 11.1			
**Weight [kg]**	131.9 ± 28.0	124.8 ± 25.6 †	91.9 ± 22.3 * ††	**<0.001**
**BMI [kg/m^2^]**	47.87 ± 6.93	45.51 ± 6.47 †	33.47 ± 6.6 * ††	**<0.001**
**WC [cm]**	125.21 ± 13.22	118.94 ± 10.23 †	97.41 ± 8.16 * ††	**<0.001**
**SAP [mmHg]**	124.4 ± 15.2	116.6 ± 12.7 †	116.0 ± 14.3 *	**<0.001**
**DAP [mmHg]**	79.3 ± 7.7	77.2 ± 6.9	76.3 ± 8.5	0.228
**Fasting glucose [mg/dL]**	103.94 ± 10.96	104.22 ± 7.71	94.61 ±16.70 * ††	**0.015**
**Insulin [pmol/L]**	20.86 ± 10.25	16.01 ± 5.71 †	5.98 ± 2.04 * ††	**<0.001**
**OGIS**	325.71 ± 44.89	344.71 ± 46.27	430.36 ± 51.12 * ††	**<0.001**
**HOMA**	5.43 ± 2.89	4.18 ± 1.74 †	1.45 ± 0.62 * ††	**<0.001**
**Resting Metabolic Rate [kcal/day]**	2226.57 ± 522.28	2034.64 ± 307.5	1737.00 ± 394.75 * ††	**<0.001**

Abbreviations: *n* = number of cases; SAP = systolic arterial pressure; DAP = diastolic arterial pressure; HR = heart rate; WC: Waist Circumference; BMI = Body Mass Index; OGIS = oral glucose insulin sensitivity index; HOMA = Homeostasis model assessment. * T2 vs. T0: *p* < 0.05; † T1 vs. T0: *p* < 0.05; †† T2 vs. T1: *p* < 0.05.

**Table 2:** Table descriptive of exercise, nutrition and stress perception variables at the three study epochs.

Variables	Time 0	Time 1	Time 2	Significance
***n***	29	29	29	
**SEDENT [h]**	33.8 ± 22.2	35.1 ± 20.8	33.5 ± 16.2	0.875
**Total Activity Volume [MET·min/week]**	59.0 ± 195.4	201.9 ± 484.2	568.8 ± 621.1 *	**0.003**
**AHA Nutrition score [AU]**	2.3 ± 1.1	2.7 ± 1.1	2.3 ± 0.8	0.282
**Stress Perception [AU]**	4.2 ± 2.9	4.4 ± 3.0	2.2 ± 2.9	**0.010**
**4SQ [AU]**	13.6 ± 10.1	11.5 ± 12.6	9.8 ± 10.0	0.117
**Tiredness Perception [AU]**	4.5 ± 3.1	3.9 ± 2.7	2.4 ± 2.9	**0.042**

Abbreviations: SEDENT = sedentariness; MET = Metabolic Equivalent; AHA score = American Heart Association Nutrition Score; 4SQ = Stress related Somatic Symptoms Questionnaire Score. * T2 vs. T0: *p* < 0.05.

**Table 3:** Representative descriptive indices of RR interval and arterial pressure variability at the three study epochs.

Variables	Time 0	Time 1	Time 2	Significance
***n***	29	29	29	
**RR [ms]**	799.9 ± 95.9	895.2 ± 132.6 †	1019.5 ± 140.7 * ††	**<0.001**
**VAR_RR_ [ms^2^]**	1053.9 ± 932.6	1860.6 ± 1681.5 †	2937.3 ± 2133.0 * ††	**<0.001**
**LFa [ms]**	362.1 ± 513.4	455.4 ± 627.5	844.6 ± 1344.2 *	**0.032**
**HFa [ms]**	231.3 ± 228.8	525.1 ± 860.6	885.0 ± 1018.8 *	**0.001**
**LF_RR_ [nu]**	49.1 ± 25.3	43.2 ± 22.3	41.3 ± 25.6	0.105
**HF_RR_ [nu]**	42.5 ±22.4	48.7 ± 22.2	53.0 ± 24.2 *	**0.023**
**RRHF [Hz]**	0.29 ± 0.06	0.30 ±0.06	0.27 ± 0.06 ††	**0.012**
**LF/HF [-]**	4.1 ± 8.5	2.8 ± 7.3	1.7 ± 2.6	0.191
**SAP_mean_ [mmHg]**	123.8 ± 19.0	116.7 ± 13.9	115.2 ± 15.7 *	**0.012**
**SAP_var_ [mmHg^2^]**	21.4 ± 17.3	18.8 ± 20.4	23.5 ± 23.3	0.636
**Alpha Index [ms/mmHg]**	10.4 ± 7.8	16.7 ± 13.0 †	23.0 ± 13.9 *	**<0.001**
**BRS**	11.2 ± 10.7	11.1 ± 7.4	28.6 ± 23.4 * ††	**0.001**
**ANSI**	33.6 ± 24.9	54.5 ± 27.7†	72.6 ± 22.9 * ††	**<0.001**

Abbreviations: RR = RR interval; VAR_RR_ = RR interval variance; LF= Low Frequency component of RR Variability; a =absolute value; HF= High Frequency component of RR Variability; nu= Normalized Unit; LF/HF= LF_RR_ on HF_RR_ ratio; SAP_mean_ = mean value of systolic arterial pressure; SAP_var_= variance of systolic arterial pressure variability; BRS: baroreceptor sensitivity; ANSI= autonomic nervous system index. * T2 vs. T0: *p* < 0.05; † T1 vs. T0: *p* < 0.05; †† T2 vs. T1: *p* < 0.05.
